# Adolescent eating behaviours: associations with autistic and ADHD traits in childhood and the mediating role of anxiety

**DOI:** 10.1111/jcpp.70051

**Published:** 2025-09-22

**Authors:** Johanna Keller, William Polmear Locke Mandy, Moritz Herle, Virginia Carter Leno

**Affiliations:** ^1^ Institute of Psychiatry, Psychology & Neuroscience King's College London London UK; ^2^ Department of Clinical Educational and Health Psychology, UCL London UK; ^3^ Centre for Brain and Cognitive Development Birkbeck, University of London London UK

**Keywords:** Autism, ADHD, eating disorder, anxiety, gender, ALSPAC

## Abstract

**Background:**

Autistic individuals and individuals with ADHD are more likely to experience eating disorders, yet the reasons for this are not well understood. We investigated whether childhood neurodivergent (i.e. autistic and ADHD) traits are associated with patterns of emotional/restrained eating and externally driven eating behaviours in adolescence, whether these associations differed by sex and if they are mediated by anxiety.

**Methods:**

We tested the association between parent‐reported childhood (age 7 years) autistic and ADHD (i.e. hyperactivity/impulsivity, inattention) traits and self‐reported adolescent (age 13) eating behaviours in a large population‐representative prospective cohort (*N* = 7,572; Avon Longitudinal Study of Parents and Children). We performed multi‐group longitudinal analysis stratified by sex to understand whether sex moderates' associations between neurodevelopmental traits and adolescent eating behaviours. Mediation models tested the extent to which observed associations were driven by mid‐childhood (age 10) anxiety symptoms. All analyses were adjusted for child sex, child ethnicity, maternal education levels and maternal age at birth.

**Results:**

Individuals who had higher childhood autistic traits were more likely to report emotional/restrained (*b* = 0.59, 95% CI [0.29, 0.88], *p* < .001, *B* = 0.07) and externally driven (*b* = 0.17, 95% CI [0.05, 0.28], *p* < .01, *B* = 0.06) eating behaviours during adolescence. Additionally, individuals with higher childhood inattention traits were more likely to report externally driven eating behaviours (*b* = 0.10, 95% CI [0.03, 0.19], *p* = .02, *B* = 0.05). No sex differences were identified in the associations. Mediation models suggested a significant indirect effect of anxiety for the association between autistic traits and emotional/restrained eating (*b* = 0.08, bootstrapped 95% CIs [0.02, 0.13]).

**Conclusions:**

Autistic and ADHD traits in childhood may share some eating behaviour phenotypes in adolescence (externally driven eating), whereas others are specific to autism (emotional/restrained eating). We present evidence for the role of anxiety in underpinning the association between autistic traits and emotional/restrained eating behaviours, suggesting an important potential intervention target.

## Introduction

Autism and ADHD are common neurodevelopmental conditions that often co‐occur and are both associated with an increased risk of experiencing disordered eating behaviours (Bleck, DeBate, & Olivardia, 2015; Inal‐Kaleli, Dogan, Kose, & Bora, 2024; Nickel et al., 2019). Understanding links between neurodevelopmental traits and disordered eating is crucial, as eating disorders are often chronic conditions associated with physical complications (Johnson, Cohen, Kasen, & Brook, 2002) and high mortality rates (Iwajomo et al., 2021). Recent evidence suggests that the prevalence of eating disorders in young people is increasing (Cybulski et al., 2021), highlighting the urgent need for evidence‐based assessment and intervention. Alterations in broader patterns of eating behaviours may be an important mechanism in the pathway between neurodevelopmental conditions and eating disorders, but how these may be altered in neurodivergent people is not well understood.

ADHD and autism are both associated with an increased likelihood of engaging in disordered eating, regardless of whether these neurodevelopmental conditions are measured at the diagnostic or trait level (Bleck et al., 2015; Martini et al., 2022; Nickel et al., 2019). For clarity on terminology, when we refer to eating disorders, we are referring to diagnosed conditions such as anorexia nervosa or binge eating disorder. However, when we refer to disordered eating, we mean patterns of eating that may not necessarily have a specific label attached to them but can have a detrimental impact on the person engaging in them. For example, this could include selective or restrictive eating that does not reach the threshold for an anorexia nervosa diagnosis. Longitudinal studies have shown that people with high autistic traits during childhood are more likely to engage in disordered eating behaviours such as purging, restricting and binge eating in adolescence (Solmi et al., 2021). Similar links have been reported between childhood ADHD and adolescent patterns of disordered eating (Levin & Rawana, 2016; Yoshimasu et al., 2012). One hypothesis is that neurodevelopmental traits may lead to altered patterns of eating, potentially influenced by the neurocognitive profiles associated with autism and ADHD. Certain patterns of eating behaviours in childhood and adolescence (e.g. fussiness in eating, food responsiveness, over‐ and undereating) have been associated with both being under‐ and overweight (Kininmonth et al., 2021; McClelland, Robinson, Potterton, Mountford, & Schmidt, 2020), and with an increased risk of developing eating disorders in adolescence (Herle et al., 2020). These early eating behaviours may therefore represent both important precursors and a potential point of intervention on the aetiological pathway. Understanding associations between these patterns of eating behaviours in childhood and autism and ADHD is necessary to inform preventative medicine efforts for neurodivergent people.

Additionally, most studies have examined the impact of autism or ADHD in isolation, despite their strong co‐occurrence (Hours, Recasens, & Baleyte, 2022). One study that considered both domains of traits, using data from the population‐based Generation R cohort, found that children with high levels of autistic traits at age 6 showed more food avoidant behaviours (increased emotional undereating, satiety responsiveness/slowness in eating and picky eating and decreased enjoyment in food) at age 10, whereas children with high levels of ADHD traits showed more food approach behaviours (increased food responsiveness and emotional overeating) (Harris, Bowling, Santos, Greaves‐Lord, & Jansen, 2022). These results suggest that although autism and ADHD are both associated with an increased risk for disordered eating, potential manifestations and mechanisms may differ between the two conditions (and associated traits).

Both autism and ADHD are more prevalent in males, although recent rates of diagnoses are rising in females (Cruz et al., 2024), largely due to improved awareness. Conversely, eating disorders are more prevalent in females (Galmiche, Déchelotte, Lambert, & Tavolacci, 2019) and gender differences are seen in sub‐clinical patterns of eating behaviours (i.e. females show more emotionally driven and restrained patterns of eating; Burton, Smit, & Lightowler, 2007). The intersection between neurodivergence and gender in terms of eating disorder outcomes is unclear. Solmi et al. (2021) observed no difference in the strength of association between autistic traits and disordered eating behaviours based on sex, whereas Barnett et al. (2021) found that this link was more pronounced in females than in males. These conflicting results may stem from differences in approaches to measuring disordered eating and sampling. Two studies found associations between hyperactivity symptoms and patterns of restrained eating were more pronounced among males than females (Grabarek & Cooper, 2008; Rastam et al., 2013); however, others report no moderating role of sex in the association between ADHD and obesity, loss of control over eating or binge eating (Egbert et al., 2018).

In associations between neurodivergence and disordered eating, anxiety may represent a key explanatory mechanism. One qualitative study found that for some autistic women, their eating disorder was an attempt to cope with their anxiety (Brede et al., 2020). A meta‐analysis identified 8 articles that found the mediating role of negative affectivity and emotional dysregulation in the relationship between ADHD and disordered eating patterns (El Archi et al., 2020), and others report that the association between autistic traits and disordered eating patterns is mediated by emotional dysregulation (Mansour et al., 2016). This is of particular relevance given the close links between negative affectivity, emotional dysregulation and anxiety (Tortella‐Feliu, Balle, & Sesé, 2010). It is well documented that anxiety symptoms and diagnoses are far higher in autistic and ADHD populations (Wu, Joubran, Kumar, Assadi, & Nguyen, 2023). Given reports of decreased effectiveness and increased unmet needs following standard interventions for eating disorders in autistic individuals (Babb et al., 2022; Kinnaird, Norton, Stewart, & Tchanturia, 2019; Tchanturia, Larsson, & Adamson, 2016), understanding the interplay between anxiety, neurodivergence and potentially maladaptive patterns of eating may illuminate mechanisms that could be targeted in more effectively tailored treatment for neurodivergent people.

In this current paper, we aim to (1) assess whether autistic traits and ADHD traits in childhood are associated with differences in eating behaviours in adolescence, (2) examine whether there are sex differences in the associations between childhood autistic and ADHD traits and adolescent eating behaviours and (3) examine whether anxiety explains (i.e. mediates) the association between childhood neurodevelopmental traits and adolescent eating behaviours.

## Methods

### Sample

We used data from the Avon Longitudinal Study of Parents and Children (ASLPAC). Pregnant women resident in Avon, UK, with expected dates of delivery between 1 April 1991 and 31 December 1992 were invited to take part in the study. The initial number of pregnancies enrolled was 14,541. Of the initial pregnancies, there was a total of 14,676 foetuses, resulting in 14,062 live births and 13,988 children who were alive at 1 year of age (Boyd et al., 2012; Fraser et al., 2013). For more details on the ALSPAC cohort, see the study website (https://www.bristol.ac.uk/alspac/). Please note that the study website contains details of all the data that is available through a fully searchable data dictionary and variable search tool (http://www.bristol.ac.uk/alspac/researchers/our‐data/).

For this study, we included participants who had complete data on both autistic and ADHD traits at age 7 and key covariates (child sex at birth, child ethnicity, maternal age at delivery, maternal education) (*N* = 7,540). For each set of twins (*n* = 203 pairs in our sample), we excluded the second‐born twin to ensure independence of the data.

Ethical approval for the study was obtained from the ALSPAC Ethics and Law Committee and the Local Research Ethics Committees. Informed consent for the use of data collected via questionnaires and clinics was obtained from participants following the recommendations of the ALSPAC Ethics and Law Committee at the time.

### Measures

#### Neurodevelopmental traits

Autistic traits were measured using the Social and Communication Disorders Checklist (SCDC), reported by their parents when their child was 7 years old. The SCDC measures social reciprocity and social communication difficulties in both clinical settings and the general population (Skuse, Mandy, & Scourfield, 2005). It is a well‐validated tool for assessing autistic traits in children and has shown to have strong internal consistency (*α* = .93) and test/re‐test reliability (*r* = .81) (Skuse et al., 2005). In the current sample, internal consistency was good (*α* = .88). The SCDC comprises 12 items rated using a 3‐point Likert scale (1 = *not true*, 2 = *quite/sometimes true* and 3 = *very/often true*). For current analyses, we used the average SCDC score, calculated from participants who had <6 items missing.

ADHD traits were measured using the Development and Well‐Being Questionnaire (DAWBA). The DAWBA is a semi‐structured diagnostic interview adapted to a questionnaire completed by the parent when the child was 7 years old. The DAWBA assesses psychopathology based on the International Classification of Disease 10th revision (ICD‐10) and the Diagnostic and Statistical Manual of Mental Disorders 4th edition (DSM‐4) (Goodman, Ford, Richards, Gatward, & Meltzer, 2000). The DAWBA has shown strong inter‐rater reliability in predicting the presence of clinical diagnoses including ADHD (Aebi et al., 2012). As previous reports suggest that different domains of ADHD symptoms may have independent associations with eating behaviours (Yilmaz et al., 2017), we examined the role of hyperactivity/impulsivity and inattention symptoms separately. The DAWBA hyperactivity/impulsivity and inattention subscales each include 9 items rated on a 3‐point Likert scale (1 = *no*, 2 = *a little more than others* and 3 = *a lot more than others*). We used the average score for each subscale, calculated from participants who had <5 items missing. In the current sample, internal consistency was excellent for both hyperactivity/impulsivity and inattention subscales (*α* =.91, .92).

#### Anxiety

An anxiety composite score was established based on the combined average of the mean symptoms (7 items, participations had to have completed at least 3 items) plus the mean severity score (6 items, participations had to have completed at least 3 items) from the DAWBA generalised anxiety items at age 10 (as used by Morales‐Muñoz, Hett, Humpston, Mallikarjun, and Marwaha (2022)). Parents were asked to report based on a 3‐point Likert scale on anxiety symptoms (1 = *not more than others*, 2 = *a little more than others* and 3 = *a lot more than others*). The DAWBA has been validated across several psychiatric disorders including anxiety symptoms (Amelio et al., 2024). In the current sample, internal consistency was acceptable (*α* = .76).

#### Eating behaviours

At age 13, adolescents self‐reported their eating behaviours using items taken from the Dutch Eating Behaviour Questionnaire (DEBQ). The questionnaire consists of three distinct subscales: external, emotional and restrained eating. These eating behaviours have been associated with the development of obesity (Kim et al., 2023) and eating disorders (Wardle, 1987) in children and adolescents. The externally driven eating subscale includes seven questions, such as ‘If food smells and looks good, do you eat more than usual?’ Responses to six of the questions were rated on a 4‐point Likert scale (*never*, *occasionally*, *sometimes* and *always*), while the seventh question included an additional response option (‘never prepare a meal’). The emotional eating subscale consists of 14 questions, including ‘Do you have a desire to eat when you feel lonely?’ Responses were rated on a 4‐point Likert scale (*yes*, *usually*, *sometimes* and *no, not at all*). Restrained eating was assessed through two questions, such as ‘Do you deliberately eat less in order not to become heavier?’ and ‘Do you eat less at mealtimes?’ Responses were rated on a 4‐point Likert scale (*yes*, *usually/frequently*, *yes, sometimes* and *never/no*). As ALSPAC included an abbreviated version of the original DEBQ items (ALSPAC included 23 questions and the original DEBQ consists of 33 questions), we conducted an exploratory factor analysis to understand whether the original three‐factor structure was replicated. We identified that a two‐factor solution was a better fit to the data, where items from the emotional and restrained eating subscales were collapsed into one factor. This is in line with reports that restrained eating and emotional eating, while measuring different behaviours, tend to co‐occur and are therefore often grouped into one domain (Burton & Abbott, 2019). Therefore, we specified a two‐factor model (emotional/restrained and externally driven eating factors) based on the pattern of factor loadings and extracted factor scores for each participant (see Supporting Information for additional details of the factor analytic approach, Figure S1 for a scree plot of Eigenvalues and Table S3 for item factor loadings).

#### Covariates

We adjusted for several potential covariates that may influence hypothesised associations. These were child sex at birth, child ethnicity, maternal education and maternal age at birth, all collected at baseline. Child ethnicity was grouped into a binary variable of white and non‐white. Maternal highest level of education was grouped into a binary variable of compulsory education and non‐compulsory education.

### Analyses

Analyses were completed in Stata 18 (StataCorp, 2023). The final script is available at https://osf.io/7fmpy/. First, structural equation models used linear regression to test the longitudinal associations between childhood neurodivergent traits and adolescent eating behaviours, while adjusting for covariates. Given that autism and ADHD often co‐occur, entering neurodevelopmental traits in the same model allowed us to understand differential effects while accounting for this known overlap (we also ran bivariate associations between autistic traits and eating behaviours, and hyperactivity/impulsivity and inattention traits and eating behaviours for completeness, see Supporting Information for results). Similarly, our analytic model set up our outcome measures of emotional/restrained and externally driven eating as correlated constructs, thereby testing the specificity of effects. Second, we tested whether sex moderated the relationship between childhood neurodevelopmental traits and adolescent eating behaviours. Here, we ran a multi‐group longitudinal analysis stratified by sex while controlling for covariates. Multi‐group models allowed all paths to differ between groups, and we tested sex‐based group differences in coefficients (i.e. parameter invariance) of interest using the Wald test. Third, we tested whether any observed associations between childhood neurodevelopmental traits and adolescent eating behaviours were in part due to increased mid‐childhood anxiety. Here, mediation models were constructed, specifying paths from neurodevelopmental traits to both mid‐childhood anxiety symptoms and adolescent eating behaviours, and paths from mid‐childhood anxiety symptoms to adolescent eating behaviours. We calculated estimates of the indirect effect of anxiety and associated 95% confidence intervals (CIs) using bootstrapping with 200 repetitions. All longitudinal models were estimated using full information maximum likelihood to account for missing data. We report both unstandardised (*b*) and standardised (*β*) coefficients. As a secondary step, we also covaried for childhood BMI, measured at the same time as neurodivergent traits. Results were substantially similar to primary analyses (see Tables S2–S4).

## Results

See Table 1 for sample descriptives.

**Table 1:** Descriptive of analytic sample

Participants' characteristics	Analytic sample	Males	Females
Mean (*SD*; range)	(*N* = 7,540)	(*n* = 3,864)	(*n* = 3,676)
Child sex (*N*, %)			
Male	3,864 (51.25%)	–	–
Female	3,676 (48.75%)	–	–
Child ethnicity (*N*, %)			
White	7,254 (96.21%)	3,721 (96.29%)	3,533 (96.11%)
Non‐white	286 (3.79%)	143 (3.71%)	143 (3.89%)
Maternal education (*N*, %)			
Compulsory	4,336 (57.51%)	2,249 (58.20%)	2,087 (56.77%)
Non‐compulsory	3,204 (42.49%)	1,615 (41.80%)	1,589 (43.23%)
Maternal age at delivery (years)	29.1 (4.52; 15–44)	29.18 (4.58; 15–44)	28.94 (4.46; 15–44)
Childhood neurodivergent traits			
Autistic traits	1.23 (0.31; 1–3)	1.27 (0.34; 1–3)	1.20 (0.26; 1–3)
Hyperactivity/impulsivity	1.27 (0.40; 1–3)	1.33 (0.45; 1–3)	1.21 (0.34; 1–3)
Inattention	1.29 (0.43; 1–3)	1.35 (0.47; 1–3)	1.22 (0.36; 1–3)

### Associations between childhood autistic and ADHD traits and adolescent eating behaviours

Multivariate models indicated a significant association between autistic traits (measured at age 7 years) and emotional/restrained eating (measured at age 14 years) (*b* = 0.59, 95% CI [0.29, 0.88], *p* < .001, *B* = 0.07) and externally driven eating (*b* = 0.17, 95% CI [0.05, 0.28], *p* < .01, *B* = 0.06). Inattention traits were not significantly associated with emotional/restrained eating (*b* = 0.20, 95% CI [−0.03, 0.43], *p* = .08, *B* = 0.04) but were associated with externally driven eating (*b* = 0.10, 95% CI [0.03, 0.19], *p* = .02, *B* = 0.05). There were no significant associations between hyperactivity/impulsivity and emotional/restrained eating (*b* = −0.22, 95% CI [−0.48, 0.04], *p* = .09, *B* = −0.04) or externally driven eating (*b* = −0.09, 95% CI [−0.19, 0.01], *p* = .06, *B* = −0.04).

### Sex as a moderator

Multi‐group models revealed no significant moderation effects of sex on the associations between childhood autistic (*p* = .78 for emotional/restrained eating, *p* = .96 for externally driven eating), hyperactivity/impulsivity (*p* = .46 for emotional/restrained eating, *p* = .53 for externally driven eating) or inattention (*p* = .83 for emotional/restrained eating, *p* = .79 for externally driven eating) traits and adolescent eating behaviours.

### Anxiety as a mediator

**Figure 1:**
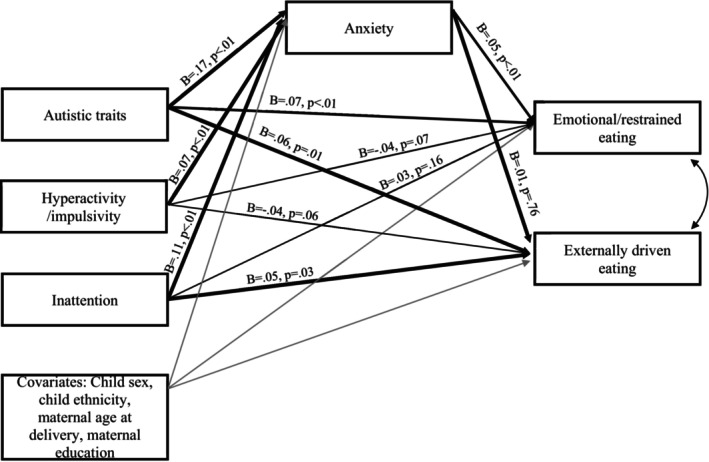
Standardised coefficients of effects in longitudinal mediation model. For brevity, covariate effects are not displayed in the figure

See Figure 1 for a graphical representation of key results and Table 2 for full results. Analyses showed a significant association between both autistic traits and anxiety (=0.15, 95% CI [0.11, 0.18], *p* < .001, *B* = 0.17) and inattention traits and anxiety (*b* = 0.07, 95% CI [0.04, 0.10], *p* = .003, *B* = 0.11), and from anxiety to emotional/restrained eating (*b* = 0.46, 95% CI [0.12, 0.81], *p* = .008, *B* = 0.05). Anxiety was not significantly associated with externally driven eating (*b* = 0.02, 95% CI [−0.11, 0.15], *p* = .76, *B* = 0.01), and thus, we calculated the indirect path from autistic traits to emotional/restrained eating (as detailed above, the path between inattention traits and emotional/restrained eating was non‐significant). Bootstrapped models found a significant indirect effect of anxiety for the association between autistic traits and emotional/restrained eating (*b* = 0.08, bootstrapped 95% CIs [0.02, 0.13]). Comparison of the indirect and the total effects suggested that around 14% of the observed association between autistic traits and adolescent emotional/restrained eating was mediated by anxiety.

**Table 2:** Results of model testing mediating role of anxiety in associations between childhood autistic, hyperactivity, inattention and adolescent eating behaviours

	*b*	*p* Value	95% CI		*B*
			Lower bound	Upper bound	
Anxiety age 10					
Autistic traits	0.15	.00	0.11	0.18	0.17
Hyperactivity/impulsivity	0.05	.00	0.02	0.08	0.07
Inattention	0.07	.00	0.04	0.10	0.11
Sex	0.04	.00	0.02	0.05	0.07
Maternal age at delivery	0.00	.59	0.00	0.00	0.01
Maternal education	0.01	.55	−0.01	0.02	0.01
Child ethnicity	0.02	.45	−0.03	0.07	0.01
Emotional/restrained eating age 13					
Mid‐childhood anxiety	0.46	.01	0.12	0.81	0.05
Autistic traits	0.53	.00	0.23	0.82	0.07
Hyperactivity/impulsivity	−0.24	.07	−0.50	0.02	−0.04
Inattention	0.17	.16	−0.06	0.39	0.03
Sex	1.79	.00	1.66	1.91	0.37
Maternal age at delivery	0.00	.95	−0.02	0.01	0.00
Maternal education	0.11	.09	−0.02	0.24	0.02
Child ethnicity	0.19	.27	−0.15	0.54	0.02
Externally driven eating age 13					
Mid‐childhood anxiety	0.02	.76	−0.11	0.15	0.01
Autistic traits	0.16	.01	0.05	0.28	0.06
Hyperactivity/impulsivity	−0.10	.06	−0.19	0.00	−0.04
Inattention	0.10	.03	0.01	0.19	0.05
Sex	0.06	.02	0.01	0.11	0.04
Maternal age at delivery	0.00	.49	0.00	0.01	0.01
Maternal education	0.13	.00	0.08	0.18	0.08
Child ethnicity	0.23	.00	0.09	0.36	0.05

## Discussion

This study evaluated longitudinal associations between childhood autistic and ADHD traits and patterns of eating behaviours during adolescence. We also investigated potential sex disparities in these associations and explored whether anxiety acts as a mediator. Results indicate that individuals with higher levels of autistic traits during childhood were more likely to engage in emotional/restrained eating and externally driven eating during adolescence. Mediation analyses suggested that anxiety accounted for a significant amount of the association between autistic traits and adolescent emotional/restrained eating behaviours. Children exhibiting higher levels of inattention traits were also more likely to engage in externally driven eating behaviours in adolescence. We did not find any association between both domains of ADHD traits and adolescent emotional/restrained eating. We found no evidence for sex differences in any of the reported associations.

Results showed that higher levels of parent‐rated autistic traits in childhood were associated with higher levels of self‐reported emotional/restrained eating in adolescence. We did not observe any associations between ADHD traits and emotional/restrained eating, suggesting some specificity of effects. This finding extends previous work reporting associations between childhood autistic traits and disordered eating in the same cohort (Carter Leno, Micali, Bryant‐Waugh, & Herle, 2022; Solmi et al., 2021). This previous work did not adjust for concurrent ADHD traits, but given associations between autistic traits and eating behaviours were present in both bivariate and multivariate models in our current analyses, this suggests previously reported associations between autism and eating behaviour outcomes are unlikely to be wholly explained by co‐occurring ADHD (and that they are present for both ‘everyday’ eating behaviours and more severe behaviours that are considered clinically relevant). This is in line with others who report that autistic but not ADHD traits are associated with later food avoidance behaviours in population‐based cohorts (Harris et al., 2022). There is evidence that engaging in emotional/restrained eating behaviours may be a risk factor for more severe patterns of disordered eating that develop into clinical diagnoses (Kim, Heo, Kang, Song, & Treasure, 2010; Reichenberger et al., 2021), suggesting a potential target for preventative medicine efforts. Psychoeducation about the potential negative consequences of restrained and emotionally driven patterns of eating, which have been shown to reduce eating disorder symptomology, may be a useful tool to offer autistic youth and their caregivers (Kurnik Mesarič, Damjanac, Debeljak, & Kodrič, 2024) and is something neurodivergent people with experience of disordered eating see as a research priority (Keller, Herle, Mandy, & Leno, 2024). A better understanding of the specific challenges experienced and how these are linked to aspects of the autism phenotype (e.g. sensory sensitivities; Brede et al., 2020) would allow interventions to be better targeted and thus more effective. In these analyses, we grouped emotional eating and restrained eating together; future work should consider measuring each type of eating behaviour separately to understand whether effects are specific to one form of eating behaviour or both.

Mediation models suggested that anxiety is one mechanism that may, in part, explain the observed association between childhood autistic traits and adolescent emotional/restrained eating. Links between anxiety and eating disorders are reported from general population cohorts (Ralph‐Nearman, Williams, Ortiz, Smith, & Levinson, 2021; Schaumberg et al., 2019); however, to our knowledge, this hypothesis has not yet been tested within the framework of neurodevelopmental conditions. One exception is a study that found emotional dysregulation mediated the link between autistic traits, body dissatisfaction and eating disorder symptoms (Mansour et al., 2016). Results of mediation analyses underscore the importance of addressing anxiety in treatment for autistic individuals with co‐occurring disordered eating patterns. It may be that patterns of emotional/restrained eating represent a strategy to attempt to bring down levels of anxiety and arousal (in line with qualitative reports from autistic adult women with restrictive eating disorders; Brede et al., 2020). Therefore, addressing high levels of anxiety (known to be more prevalent in autistic youth; Hollocks et al., 2023; Simonoff et al., 2008) may improve treatment outcomes for autistic people seeking support with disordered eating. Although interventions that target the link between symptoms of anxiety and disordered eating have been proposed for the general population (Levinson, Cusack, Brown, & Smith, 2022), given reports of decreased effectiveness and increased unmet needs following mainstream interventions for autistic individuals (Babb et al., 2022; Kinnaird et al., 2019; Tchanturia et al., 2016), any new interventions must be adapted in collaboration with members of the neurodivergent community who have lived experience of disordered eating. This will ensure that the content and delivery of treatment are accessible and acceptable to neurodivergent people (and thus improve effectiveness). An experience‐based co‐design approach has been used to successfully adapt interventions for anxiety disorders in autistic children (Cullingham et al., 2025); a similar process could also be used for interventions targeting anxiety symptoms in the context of disordered eating.

However, it should be noted that mid‐childhood anxiety did not completely explain observed associations between autistic traits and restrained/emotional eating behaviours (analyses estimated around 14% of the variance was accounted for by anxiety). Therefore, more research is needed to understand the other mechanisms that may be present. Furthermore, given reports of differential associations between subdomains of anxiety symptoms and different types of disordered eating (Schaumberg et al., 2019), it is also important for future studies to investigate whether other domains of anxiety (e.g. social phobia, OCD) act as mediators in a similar manner. Finally, it may be that other unobserved factors could better explain the overlap between autistic traits, anxiety and eating behaviours. For example, they may share a genetic aetiology (Capusan et al., 2017; Yao et al., 2019). Genetically informed studies are needed to understand the extent to which our reported directional effects are truly causal.

Results also showed that higher levels of childhood inattention and autistic traits were associated with greater externally driven eating behaviours in adolescence. Interestingly, although no significant associations were found with impulsivity/hyperactivity, we note the directionality of effects was the opposite to that observed for autistic and inattention traits. This pattern of results suggests that different domains of ADHD traits may have differential developmental impacts on eating behaviours. Current results contrast with previous reports that children displaying combined inattention and hyperactivity/impulsivity traits were more predisposed to engaging in disordered eating behaviours during late adolescence compared to those with predominantly inattention or hyperactivity/impulsivity traits in isolation (Yilmaz et al., 2017). Dufour et al. (2023) found that higher hyperactivity traits at age three were associated with elevated levels of eating disorder symptoms at age 12, which contrasts to our lack of observed associations with hyperactivity/impulsivity traits. One way to reconcile these differences is to consider that most studies do not include autistic and ADHD characteristics within the same explanatory model, despite their high co‐occurrence rates (Ronald, Larsson, Anckarsäter, & Lichtenstein, 2014; Ronald, Simonoff, Kuntsi, Asherson, & Plomin, 2008; Simonoff et al., 2008).

The association between inattentiveness and externally driven eating behaviours can be viewed as in keeping with reports of a link between ADHD and out of control eating (Nickel et al., 2019), as well as binge eating (Bleck et al., 2015; Cortese, Bernardina, & Mouren, 2007; Sonneville et al., 2015). A study that used a Dutch population‐representative cohort by Harris et al. (2022), which accounted for the co‐occurrence of autism and ADHD, found children with high ADHD traits were generally characterised by food approach behaviours, and that these food approach behaviours (food responsiveness and emotional eating) could in part explain the association between ADHD traits and increased adolescent BMI, underscoring the potential health consequences of ADHD‐associated patterns of eating.

Current analyses revealed no significant sex differences in the association between childhood autistic and ADHD traits and eating behaviours in adolescence. Dufour et al. (2023) also report no sex differences when investigating the link between impulsivity and disordered eating, and Solmi et al. (2021) reported that the association between childhood autistic traits and disordered eating appeared to be consistent across both girls and boys. However, Solmi and colleagues noted a low prevalence of disordered eating among boys in their study, potentially limiting their statistical power. In the current study (where we use the same cohort as Solmi et al), we focused on less severe eating behaviours, which theoretically should have led to a less skewed distribution of scores. However, it could still be the case that despite the large sample size, we had insufficient power to detect sex moderation effects. However, others have found that associations between childhood autistic traits and adolescent disordered eating are stronger (Barnett et al., 2021) or only present (van 't Hof et al., 2020) in females. Whereas Harris and colleagues found that the associations between enjoyment of food and autistic traits were only present in males (Harris et al., 2022). These divergent findings may reflect both differences in statistical power across studies (which will be in part driven by the sex ratio of the sample), but also gendered expectations about the presentation of autism, ADHD and disordered eating, a phenomenon increasingly recognised in research on neurodivergence and eating disorders (Barnett et al., 2021). It is possible that data collected decades ago (such as in many of the currently available longitudinal cohort datasets, including that used in the current analyses) used measurement tools that significantly underestimate neurodivergent traits in females, which would decrease statistical power to detect moderation effects. This would be in line with research suggesting that there is a diagnostic gender bias in favour of male presentations of autism, leading to underdiagnosis in females (Loomes, Hull, & Mandy, 2017). This may in part explain the discrepancy between the clinical observation that severe restrictive eating patterns are significantly more common in women, including autistic women and the lack of sex effects currently observed.

### Strengths and limitations

Strengths of this current work include the use of a well‐characterised longitudinal dataset, with a large sample size and a relatively even representation of males and females. To our knowledge, very few studies have considered the developmental impacts of *both* autism and ADHD on eating behaviours. Including both domains of traits in the same analytic model allowed us not only to understand the specificity of any longitudinal associations but also to gain confidence that unmeasured ADHD traits were not driving the observed autism‐eating behaviour associations, and vice versa.

However, there are limitations to this work. Our measures for childhood autistic, ADHD traits and anxiety levels were completed by the primary parent (usually the mother) of the child. Studies suggest several factors can influence a mother's perception of their child; for example, the mother's own emotional difficulties (Najman et al., 2001). Additionally, there were only two available items that captured restrained eating; future studies would benefit from greater coverage. Also, there may be other forms of eating behaviours that are relevant to diagnoses aside from anorexia nervosa (e.g. binge eating disorder, bulimia nervosa) that should also be considered in future work. A further limitation is the overwhelming representation of individuals from a white ethnic background (96%) in the ALSPAC cohort. This raises concerns regarding the generalisability of our findings. Cultural norms, attitudes and beliefs impact the development of eating disorders (Miller & Pumariega, 2001), so results may be specific to the cultural context from which they were drawn (a country with relatively high levels of economic growth and security). Conducting similar studies in different geographical locations with more representative samples will provide valuable cross‐cultural perspectives and augment the effectiveness of future intervention strategies.

## Conclusions

Our findings shed light on the nature of associations between childhood autistic and ADHD traits and later eating behaviours during adolescence, which may explain the increased prevalence of eating disorders in these populations. Results suggest that autistic and ADHD traits may share associations with certain eating behaviour phenotypes (externally driven eating), whereas others are more specific to autism (emotional/restrained eating). Anxiety may mediate the relationship between autistic traits and emotional/restrained eating, suggesting an important potential intervention target. This study underscores the need to raise awareness of potentially maladaptive patterns of eating in neurodivergent children and develop evidence‐based support to promote positive adolescent well‐being and health outcomes.

## Ethical considerations

Ethical approval for the study was obtained from the ALSPAC Ethics and Law Committee and the Local Research Ethics Committees. Informed consent for the use of data collected via questionnaires and clinics was obtained from participants following the recommendations of the ALSPAC Ethics and Law Committee at the time. ALSPAC was initially approved by the Bristol and Weston Health Authority (E1808 Children of the Nineties: Avon Longitudinal Study of Pregnancy and Childhood (ALSPAC) (28 November 1989)), the Southmead Health Authority (49/89 Children of the Nineties – ‘ALSPAC’ (5 April 1990)) and the Frenchay Health Authority (90/8 Children of the Nineties (28 June 1990)). See https://www.bristol.ac.uk/alspac/researchers/research‐ethics/ for details on ethical approval at specific waves of data collection.

**Key points**

**What's known?**

Autism and ADHD are associated with an increased risk for experiencing disordered eating; however, the mechanisms that underpin this risk are not well understood.

**What's new?**

Using a large, population‐based cohort, we show that autistic traits in childhood are associated with emotional/restrained and externally driven eating behaviours in adolescence. ADHD traits in childhood were also associated with externally driven eating behaviours. Longitudinal associations were not moderated by sex, but the association between autistic traits and emotional/restrained eating was partially mediated by mid‐childhood anxiety symptoms.

**What's relevant?**

Anxiety may be an important area of intervention to prevent later disordered eating in autistic youth.

## Supporting information


**Table S1.** Factor analysis of abbreviated items from the Dutch Eating Behaviour Questionnaire in ALSPAC.
**Figure S1.** Scree plot for item factor analysis of Eating Behaviour Questionnaire items.
**Table S2.** Associations between childhood autistic, hyperactivity, inattention and adolescent eating behaviours, adjusting for childhood BMI.
**Table S3.** Invariance of associations between childhood autistic, hyperactivity, inattention and adolescent eating behaviours by sex, adjusting for childhood BMI.
**Table S4.** Model testing mediating role of anxiety in associations between childhood autistic, hyperactivity, inattention and adolescent eating behaviours, adjusting for childhood BMI.

## Data Availability

VCL and MH confirm that they had full access to all the data in the study and take responsibility for the integrity of the data and the accuracy of the data analysis. Data are available through application via the ALSPAC website (https://www.bristol.ac.uk/alspac/).
