# Supplementary material for: Adolescent eating behaviours: associations with autistic and ADHD traits in childhood and the mediating role of anxiety
Source: J Child Psychol Psychiatry. 2025 Sep 22;67(3):344–54. doi: 10.1111/jcpp.70051 (PMC12883572; doi:10.1111/jcpp.70051)
Supplement: Supplementary file 1 — Table S1. Factor analysis of abbreviated items from the Dutch Eating Behaviour Questionnaire in ALSPAC. Figure S1. Scree plot for item factor analysis of Eating Behaviour Questionnaire items. Table S2. Associations between childhood autistic, hyperactivity, inattention and adolescent eating behaviours, adjusting for childhood BMI. Table S3. Invariance of associations between childhood autistic, hyperactivity, inattention and adolescent eating behaviours by sex, adjusting for childhood BMI. Table S4. Model testing mediating role of anxiety in associations between childhood autistic, hyperactivity, inattention and adolescent eating behaviours, adjusting for childhood BMI. [file JCPP-67-344-s001.docx]

**Adolescent eating behaviours: associations with autistic and ADHD traits in childhood and the mediating role of anxiety**

**Supporting Information**

Factor analysis for eating behaviours

We performed an exploratory Item Factor Analysis (IFA) to investigate whether the sub-set of items on the Dutch Eating Behaviour questionnaire utilised in the ALSPAC formed the three underlying factors found in the unabridged version of the questionnaire. Bartlett’s test of sphericity was significant (χ^2^ = 48468.35, p<.001) and the Kaiser-Meyer-Olkin Measure of Sampling Adequacy was equal to .916, both suggesting the data was appropriate for factor analysis. Inspection of the scree plot (see Figure S1) and eigenvalues suggested a two factor solution was the most appropriate fit to the data, with items on the emotional and restraint eating largely loading on the same factor (see Table S1). We therefore ran a confirmatory factor analysis based on the eIFA results and extracted the factor scores. Factor scores were set as missing if any of the individual questionnaire items were missing from the specified subscale.

*Table S1. Factor analysis of Abbreviated Items from the Dutch Eating Behaviour Questionnaire in ALSPAC*

| Variable | Standardised Loading  Factor 1 | Standardised Loading  Factor 2 |
| --- | --- | --- |
| Eating less at mealtimes | 0.61 |  |
| Refusing food and drinks due to weight concerns | 0.62 |  |
| Eating more than usual when irritated | 0.75 |  |
| Eating more than usual when there is nothing to do | 0.47 |  |
| Eating more than usual when depressed or discouraged | 0.89 |  |
| Eating more than usual when feeling lonely | 0.78 |  |
| Eating more than usual when being let down | 0.85 |  |
| Eating more than usual when feeling happy. |  | 0.59 |
| Eating more than usual when something unpleasant is due to happen | 0.82 |  |
| Eating more than usual when feeling anxious, worried, or tense | 0.79 |  |
| Eating more than usual when things have gone wrong. | 0.89 |  |
| Eating more than usual when frightened | 0.74 |  |
| Eating more than usual when disappointed | 0.82 |  |
| Eating more than usual when feeling upset | 0.91 |  |
| Eating more than usual when restless or bored | 0.46 |  |
| Excitement causing increased food intake |  | 0.57 |
| Eating more than usual when food taste good |  | 0.78 |
| Eating more than usual because of food’s smell and looks |  | 0.78 |
| Smell and sight causing a desire to eat |  | 0.74 |
| Other people eating causes a desire to eat |  | 0.73 |
| Resisting delicious foods |  |  |
| Increased food intake if others are eating |  | 0.59 |
| Wanting to eat when preparing a meal |  | 0.49 |

**Note**: We summarise the content of the items here but do not give exact wording to respect the developer’s copyright. Blank represents loadings of <0.4

Figure S1. Screeplot for Item Factor Analysis of Eating Behaviour Questionnaire Items.

Bivariate associations between autistic traits and eating behaviours.

Bivariate analyses indicate a significant positive association between autistic traits and emotional/restrained eating behaviours. Participants with higher levels of autistic traits during childhood were more likely to report emotional/restrained eating behaviours in adolescence (b$=0.56, 95\% CIs=\left[ 0.33,0.78 \right], p<0.001, \beta=0.71)$. A significant positive association was also observed between childhood autistic traits and externally driven eating behaviours in adolescence ($b=0.17, 95\%CI \left[ 0.08,0.25 \right], p<0.001, B=0.06)$.

Bivariate associations between ADHD traits and eating behaviours.

Bivariate analyses showed higher levels of childhood inattention traits were associated with higher levels of emotional/restrained eating behaviours during adolescence ($b=0.29, 95\%CI \left[ 0.07,0.52 \right], p=0.01, B=0.05$). Additionally, higher levels of childhood inattention were also associated with higher levels of externally driven eating behaviours in adolescence ($b=0.13, 95\%CI [0.04,0.21], p=0.003, B=0.06)$. No significant associations were found between impulsivity/hyperactivity and adolescent emotional/restrained ($b=-0.01, 95\%CI \left[ -0.25,0.22 \right], p=0.904, B=-0.002)$ or external ($b=-0.04, 95\%CI\left[ -0.13,0.06 \right], p=0.440,B=-0.02$) eating behaviours.

*Table S2. Associations between Childhood Autistic, Hyperactivity, Inattention and Adolescent Eating Behaviours, Adjusting for Childhood BMI*

|  |  |  | **95% CI** | |  |
| --- | --- | --- | --- | --- | --- |
|  | **b** | **p value** | **Lower bound** | **Upper bound** | **B** |
| **Emotional/restrained Eating Age 13** | | | | | |
| Autistic traits | 0.56 | 0.00 | 0.27 | 0.86 | 0.07 |
| Hyperactivity/impulsivity | -0.21 | 0.10 | -0.47 | 0.04 | -0.04 |
| Inattention | 0.21 | 0.07 | -0.02 | 0.43 | 0.04 |
| Sex | 1.78 | 0.00 | 1.65 | 1.90 | 0.37 |
| Maternal age at delivery | 0.00 | 0.95 | -0.01 | 0.02 | 0.00 |
| Maternal education | 0.13 | 0.05 | 0.00 | 0.26 | 0.03 |
| Child ethnicity | 0.21 | 0.24 | -0.14 | 0.55 | 0.02 |
| Age 7 BMI | 0.11 | 0.00 | 0.08 | 0.14 | 0.09 |
| **Externally Driven Eating Age 13** | | | | | |
| Autistic traits | 0.17 | 0.00 | 0.06 | 0.28 | 0.06 |
| Hyperactivity/impulsivity | -0.10 | 0.06 | -0.20 | 0.00 | -0.04 |
| Inattention | 0.10 | 0.02 | 0.01 | 0.19 | 0.05 |
| Sex | 0.07 | 0.01 | 0.02 | 0.12 | 0.04 |
| Maternal age at delivery | 0.00 | 0.52 | 0.00 | 0.01 | 0.01 |
| Maternal education | 0.13 | 0.00 | 0.08 | 0.18 | 0.07 |
| Child ethnicity | 0.23 | 0.00 | 0.09 | 0.36 | 0.05 |
| Age 7 BMI | -0.02 | 0.00 | -0.03 | -0.01 | -0.05 |

*Table S3. Invariance of Associations between Childhood Autistic, Hyperactivity, Inattention and Adolescent Eating Behaviours by Sex, Adjusting for Childhood BMI*

| **Emotional/restrained Eating Age 13** | **Chi^2^** | **p value** |
| --- | --- | --- |
| Autistic traits | 0.12 | 0.735 |
| Hyperactivity/impulsivity | 0.49 | 0.486 |
| Inattention | 0.01 | 0.978 |
| **Externally Driven Eating Age 13** | **B** | **p value** |
| Autistic traits | 0.01 | 0.993 |
| Hyperactivity/impulsivity | 0.47 | 0.495 |
| Inattention | 0.10 | 0.747 |

*Table S4. Model Testing Mediating Role of Anxiety in Associations between Childhood Autistic, Hyperactivity, Inattention and Adolescent Eating Behaviours, Adjusting for Childhood BMI*

|  |  |  | **95% CI** | |  |
| --- | --- | --- | --- | --- | --- |
|  | **b** | **p value** | **Lower bound** | **Upper bound** | **B** |
| **Anxiety Age 10** | | | | | |
| Autistic traits | 0.15 | 0.00 | 0.11 | 0.18 | 0.17 |
| Hyperactivity/impulsivity | 0.05 | 0.00 | 0.02 | 0.08 | 0.07 |
| Inattention | 0.07 | 0.00 | 0.04 | 0.10 | 0.11 |
| Sex | 0.04 | 0.00 | 0.02 | 0.05 | 0.07 |
| Maternal age at delivery | 0.00 | 0.60 | 0.00 | 0.00 | 0.01 |
| Maternal education | 0.01 | 0.54 | -0.01 | 0.02 | 0.01 |
| Child ethnicity | 0.02 | 0.45 | -0.03 | 0.07 | 0.01 |
| Age 7 BMI | 0.00 | 0.73 | -0.01 | 0.00 | -0.01 |
| **Emotional/restrained Eating Age 13** | | | | | |
| Mid-Childhood Anxiety | 0.48 | 0.01 | 0.14 | 0.83 | 0.05 |
| Autistic traits | 0.50 | 0.00 | 0.20 | 0.80 | 0.06 |
| Hyperactivity/impulsivity | -0.23 | 0.08 | -0.49 | 0.02 | -0.04 |
| Inattention | 0.17 | 0.14 | -0.06 | 0.40 | 0.03 |
| Sex | 1.76 | 0.00 | 1.63 | 1.89 | 0.37 |
| Maternal age at delivery | 0.00 | 0.95 | -0.01 | 0.02 | 0.00 |
| Maternal education | 0.13 | 0.06 | 0.00 | 0.26 | 0.03 |
| Child ethnicity | 0.20 | 0.26 | -0.14 | 0.54 | 0.02 |
| Age 7 BMI | 0.11 | 0.00 | 0.08 | 0.14 | 0.09 |
| **Externally Driven Eating Age 13** | | | | | |
| Mid-Childhood Anxiety | 0.02 | 0.78 | -0.11 | 0.15 | 0.01 |
| Autistic traits | 0.17 | 0.00 | 0.05 | 0.28 | 0.06 |
| Hyperactivity/impulsivity | -0.10 | 0.06 | -0.20 | 0.00 | -0.05 |
| Inattention | 0.10 | 0.03 | 0.01 | 0.19 | 0.05 |
| Sex | 0.07 | 0.01 | 0.02 | 0.12 | 0.04 |
| Maternal age at delivery | 0.00 | 0.52 | 0.00 | 0.01 | 0.01 |
| Maternal education | 0.13 | 0.00 | 0.08 | 0.18 | 0.07 |
| Child ethnicity | 0.23 | 0.00 | 0.09 | 0.36 | 0.05 |
| Age 7 BMI | -0.02 | 0.00 | -0.03 | -0.01 | -0.05 |

Bootstrapped models found a significant indirect effect of anxiety for the association between autistic traits and emotional/restrained eating (b = 0.07, bootstrapped 95% CIs [.02,.13]).
